# Rural Aging: Multidisciplinary, Multinational Innovations That Support New Approaches to Address Unmet Needs

**DOI:** 10.1093/geroni/igaa057.2089

**Published:** 2020-12-16

**Authors:** Roger O’Sullivan, Lyn Holley, Megan Bond

## Abstract

The meaning of aging in rural areas is not well explored in the literature. To address this gap each presenter in this multidisciplinary and international group of researchers will address different components of rural ageing. An exact definition of “rural” is essential to forming, implementing or evaluating policies and programs impacting rural populations; however there is no universal definition. Cohen introduces definitional issues, and underscores the heterogeneity and regional variability of “rurality” and how such factors drive rural-urban disparities. Pendergrast, an early career researcher, reports results of in-depth semi-structured interviews that examined institutional influences of social networks on health of rural older adults, with specific focus on support services. Leavey describes lessons learned from the activities of PLACE-EE, a transnational partnership of public health agencies, local authorities, academics and ICT experts dedicated to improving the quality of life for older people. He describes the impacts on relationships of a program of community engagement and intergenerational exchange between younger and older citizens that used an assets-based approach. Żurek explores the potential for reward structures that can be embedded in ‘social games’ to motivate older adults to exercise in the context of research conducted in the rural US (Appalachia) and urban Poland (Krakow), and suggests a new direction that can inform services intended to improve health and happiness of rural adults. Our discussant will reflect on the major themes that emerge from these multidisciplinary perspectives, especially the potential for intersection of rural community-based innovations and learning from different regions of the world. Rural Aging Interest Group Sponsored Symposium.

